# Structural basis of DNA recognition by PCG2 reveals a novel DNA binding mode for winged helix-turn-helix domains

**DOI:** 10.1093/nar/gku1351

**Published:** 2014-12-29

**Authors:** Junfeng Liu, Jinguang Huang, Yanxiang Zhao, Huaian Liu, Dawei Wang, Jun Yang, Wensheng Zhao, Ian A. Taylor, You-Liang Peng

**Affiliations:** 1 MOA Key Laboratory of Plant Pathology, China Agricultural University, Beijing 100193, China; 2 State key Laboratory of Agrobiotechnology, China Agricultural University, Beijing 100193, China; 3 College of Agronomy and Plant Protection, Qingdao Agricultural University, Qingdao, Shandong 266109, China; 4 Division of Molecular Structure, MRC-NIMR, London, NW7 1AA, UK

## Abstract

The MBP1 family proteins are the DNA binding subunits of MBF cell-cycle transcription factor complexes and contain an N terminal winged helix-turn-helix (wHTH) DNA binding domain (DBD). Although the DNA binding mechanism of MBP1 from *Saccharomyces cerevisiae* has been extensively studied, the structural framework and the DNA binding mode of other MBP1 family proteins remains to be disclosed. Here, we determined the crystal structure of the DBD of PCG2, the *Magnaporthe oryzae* orthologue of MBP1, bound to MCB–DNA. The structure revealed that the wing, the 20-loop, helix A and helix B in PCG2–DBD are important elements for DNA binding. Unlike previously characterized wHTH proteins, PCG2–DBD utilizes the wing and helix-B to bind the minor groove and the major groove of the MCB–DNA whilst the 20-loop and helix A interact non-specifically with DNA. Notably, two glutamines Q89 and Q82 within the wing were found to recognize the MCB core CGCG sequence through making hydrogen bond interactions. Further *in vitro* assays confirmed essential roles of Q89 and Q82 in the DNA binding. These data together indicate that the MBP1 homologue PCG2 employs an unusual mode of binding to target DNA and demonstrate the versatility of wHTH domains.

## INTRODUCTION

The START point, also referred to as the restriction checkpoint in the mammalian cell cycle, is the period between late G1 to S in the cell cycle (1–3). During this stage, many genes required for DNA replication are induced in preparation for DNA synthesis in the S-phase. In the mammalian cell cycle, heterodimeric E2F/DP complexes regulate transcription at the restriction checkpoint (4). In comparison, transcription of the START genes in *Saccharomyces cerevisiae* is controlled by the MBF (MCB-Binding Factor) and SBF (SCB-Binding Factor) transcription factor complexes (1–3) and by the two functionally homologous complexes, Res1/Cdc10 and Res2/Cdc10 in the fission yeast *Schizosaccharomyces pombe* (5–9).

In the yeast heteromeric transcription factor complexes, a common subunit such as Swi6 or Cdc10 is required for transcription activation. However, these subunits lack any DNA binding activity (2,10 and 11). Instead, DNA recognition is provided by the other subunit of the MBF and SBF complexes. In *S. cerevisiae*, Swi6 combines with MBP1 to form MBF and with Swi4 to form SBF (1). In *S. pombe*, Res1 or Res2 provides the DNA binding activity for the Res1/Cdc10 and Res2/Cdc10 protein complexes, respectively (7). The four DNA binding proteins, MBP1, Swi4, Res1 and Res2 all have the same arrangement of domains, each consisting of an N-terminal DNA binding domain (DBD), a C-terminal heteromerization domain and a central ANK(Ankyrin) repeat region (11). In each of the four proteins, the N-terminal DBD is responsible for recognizing different DNA sequences. Swi4 binds to the SCB (Swi6/4 dependent cell cycle box) motif, 5′-CACGAAA-3′, while MBP1, Res1 and Res2 binding to the MCB (*Mlu* I cell cycle box) sequences with consensus 5′-ACGCGTNA-3′.

In the past decade, a number of studies have characterized the interaction between MBP1 and MCB–DNA sequences (12–20). The structure of the DBD of MBP1 has been determined by both X-ray crystallography and nuclear magnetic resonance (NMR) methods and revealed that MBP1–DBD has the same topology as the winged helix-turn-helix (wHTH) domain (12,13, and 17), although they share very low amino acid sequence identity (15). To date, two DNA binding modes for wHTH domain have been reported in accordance with the crystal structures of wHTH–DNA complexes. These include the transcription factors CAP (21), HNF3γ (22), ETS (23), E2F2/DP2 (4), RFX1 (24) and the LexA repressor (25). For all of these proteins except RFX1, the ‘recognition helices’ in the DBDs are the key elements that interact with the major or minor grooves of the DNA. RFX1 has an atypical binding module in the DNA complex, in which the wing instead of helix B recognizes the DNA. The wing region and helix B in the DBD of MBP1 were also predicted to interact with DNA (15,20). However, the molecular details for the interactions between the DNA binding elements of MBP1 family proteins and DNA remain unknown.

To clarify whether the DNA binding mode of MBP1 and its orthologues is similar to previously characterized wHTH domain, we have determined the crystal structure of the DBD of PCG2, the orthologue of MBP1 in the rice blast fungus *Magnaporthe oryzae* (26), bound to an MCB–DNA motif. The structure reveals that the wing (80-loop), helix B, the 20-loop and helix A are important for MCB–DNA binding and notably, that the wing and helix B recognize the minor groove and major groove of the DNA respectively. In addition, the 20-loop and helix A, two secondary elements that were not predicted in the previous reports to have DNA-binding activity, were found to non-specifically interact with the DNA in the protein–DNA interface. Further, within the wing, a conserved 82–89 QXGXGXXQ motif recognizes the core CGCG sequence of MCB–DNA through hydrogen bond interactions. By mutational analysis and *in vitro* DNA binding assays, we demonstrate essential roles of key residues in the conserved 82–89 QXGXGXXQ motif in the DNA-binding and show that the C terminus of PCG2–DBD is important for the DNA binding. Taken together, our data reveal that the MBP1 homologue PCG2 utilizes a novel DNA-binding mode that is distinct from those identified in other proteins with wHTH domain.

## MATERIALS AND METHODS

### Cloning, mutation, expression and purification

DNA fragments encoding PCG2 (1–138, 1–128, 12–138 or 12–128) were cloned from the full length ORF (Open Reading Frame) of PCG2 by polymerase chain reaction (PCR). To construct the expression vectors, the resulting PCR products were inserted between the NcoI and XhoI restriction sites of the pHAT2 vector (kindly supplied by Dr Arie Geerlof, EMBO) to produce N-terminally 6xhistidine tagged fusion proteins. Single point mutagenesis was performed according to the Fast Mutagenesis System (Beijing TransGen Biotech Co., Ltd) with a recombinant plasmid of PCG2 (1–138). For expression in *Escherichia coli*, competent cells of strain BL21 (DE3) were transformed with the recombinant vectors described above. Cells were grown at 310K in LB medium that contained 100 μg ml^−1^ of antibiotics until the OD600 reached 0.5. expression of the protein was induced by addition of 0.1 mM IPTG (Isopropyl-β-D-Thiogalactopyranoside) and overnight incubation at 289K. The *E. coli* cells were harvested by centrifugation and lysed by sonication. Proteins were purified with Ni-Chelating Sepharose™ Fast Flow Agarose, followed by ion exchange on Resource™ Q and finally by gel filtration using a Superdex™ 200 10/300 GL (GE Healthcare Co.) following the instructions provided by the manufacturers. Purified proteins were concentrated to a final concentration of 10 mg ml^−1^ with an Amicon Ultra-15 centrifugal filter with a 5-kDa molecular-weight cut-off value (Millipore).

### Circular dichroism analysis

Circular dichroism (CD) measurements of purified recombinant wild-type and mutant PCG2–DBDs were performed on a Chirascan-plus spectropolarimeter (Applied Photophysics, Leatherhead, UK) at room temperature using a quartz cell with a path length of 1 mm. Protein samples were prepared in 10 mM PBS pH 7.0 at a concentration of ∼0.2 mg ml^−1^. Spectra were recorded from 195 to 260 nm at a scan speed of 60nm/min and averaged from three replicates.

### DNA binding analysis by surface plasmon resonance

**Figure 1.**
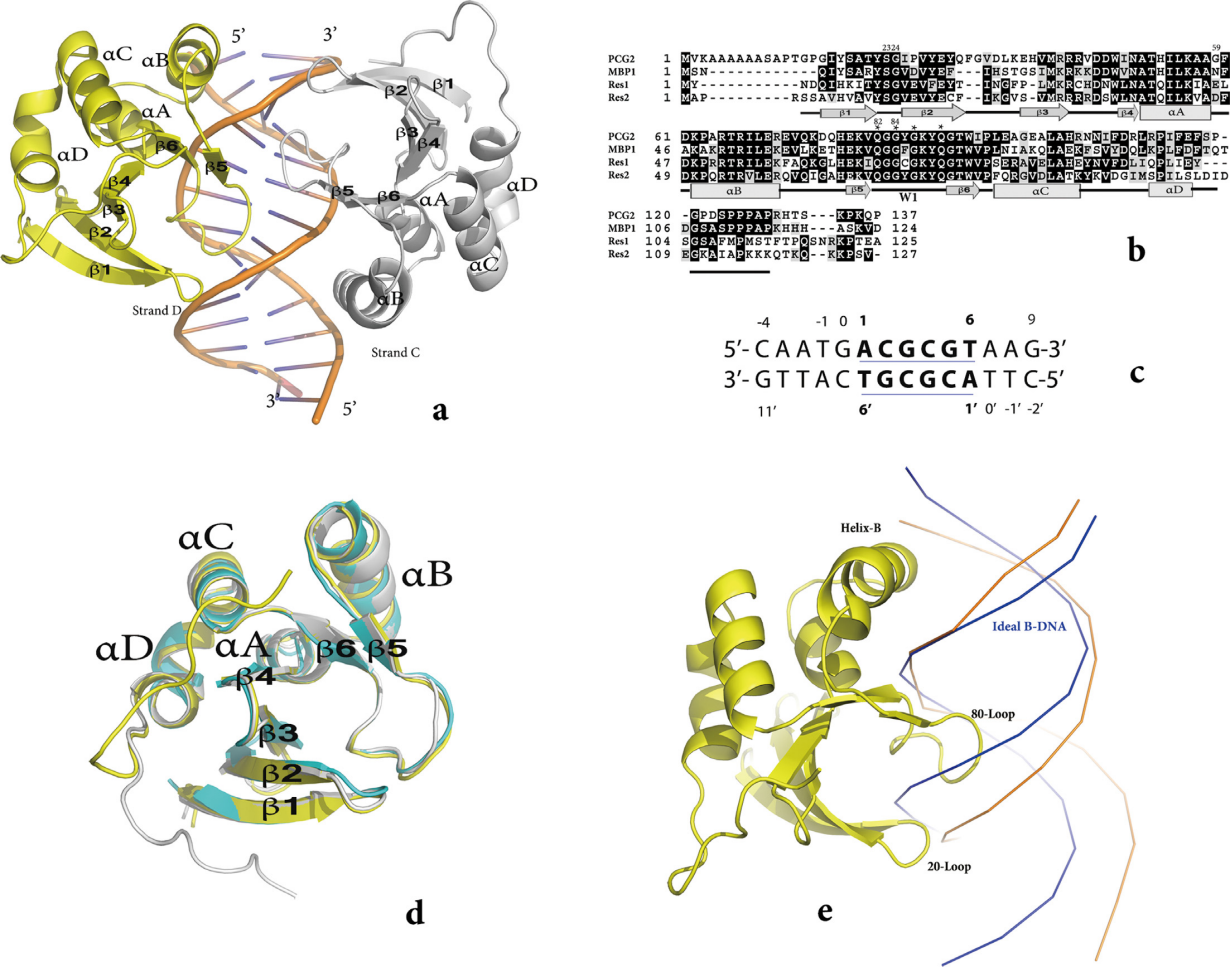
The overall structure of the PCG2–DBD–DNA complex. (**a**) The asymmetric unit. The crystal structure of the complex consists of two monomers of PCG2–DBD, Monomer-A (yellow), -B (grey) and one molecule of MCB–DNA (strands C and D). Protein secondary structure elements are labelled β1 –β6 and αA–αD and DNA stand termni labelled 5′ and 3′. (**b**) Sequence alignment of DBDs of MBP1 from the budding yeast, Res1 and Res2 from the fission yeast and PCG2 from the rice blast fungus. Conserved residues are highlighted in black. Key residues for DNA recognition are labelled with stars. (**c**) The sequence of the DNA duplex used in the co-crystallization. The MCB-box site is underlined in bold. Strand C is numbered from 1 to 6 and Strand D 1′ to 6′. (**d**) Superposition of the structures of monomer-A (yellow) and B (grey) of PCG2–DBD and MBP1–DBD (blue). (**e**) A comparison of the backbone conformation of ideal B-DNA (blue) and that of MCB–DNA in the complex structure (orange) showing changes in width of the major and minor grooves.

The DNA binding affinity of the wild-type and mutant PCG2–DBD was monitored by surface plasmon resonance (SPR) using a Biacore T100 system (GE Health Sciences). Complementary oligonucleotides (MCB1, Figure 1c), including one strand with a 5′-biotin-label (5′-CTTACGCGTCATTG-3′), were annealed and captured on a streptavidin binding CM5 sensor chip (102 response units). The running buffer contained 20 mM Tris (pH 8.0), 150 mM NaCl and 0.005% (v/v) Tween20. A blank flow cell was used as a reference. The wild-type and mutant PCG2–DBD proteins were prepared at different concentrations by step-wise dilution in the running buffer and injected over the DNA surface and blank flow cell for 1 min at a flow rate of 30 μl min^−1^. Duplicate measurements were recorded. Data were analysed with the Biacore T100 evaluation software. Equilibrium association constants were calculated using a steady state affinity model.

### Protein–DNA complex preparation

All DNA oligonucleotides were synthesized and further purified with PAGE by the Shanghai Sangon Biotechnology Company (Shanghai, China). DNA concentrations were determined from UV absorbance at 260 nm. The DNA duplexes used for crystallization and SPR experiments were generated by heating the mixture of complementary oligonucleotides at 95°C for 5 min and slowly cooling to 25°C over 1 h in the annealing buffer of 20 mM Tris–HCl (pH 8.0). Prior to gel filtration chromatography, PCG2–DBD and the double-stranded MCB1–DNA were mixed and incubated at 4°C for 12 h. The DNA–protein complex was then purified by gel filtration chromatography with a Superdex™ 200 10/300 GL column (GE Healthcare) in a buffer containing 20 mM Tris (pH 8.0) and 150 mM NaCl.

### Analytical ultracentrifugation

Sedimentation velocity analytical ultracentrifugation (SV-AUC) was carried out in a BECKMAN COULTER XL-I analytical ultra-centrifuge. PCG2–DBD–DNA complex was prepared by mixing 1, 5 or 10 mg ml^−1^ protein with MCB–DNA at the ratio 2:1 in a buffer of 20 mM Tris–HCl (pH 8.0) and 150 mM NaCl. Samples were centrifuged at 40 000 rpm in an An-50Ti rotor at 293K. SV-AUC Data collected using interference optics were analysed in terms of the size distribution functions C(S) using the program of the SEDFIT (27).

### Crystallization

Initial PCG2–DBD–DNA complex crystals were obtained by the sitting drop vapour diffusion method from a PEGRx and PEG/ION crystallization screen (Hampton Research Co.) dispensed with an Oryx4 crystallization robot (Douglas Instruments Ltd). A mixture of 0.15 μl protein and equal volume of well solution was equilibrated against 73 μl of reservoir solution. Crystals appeared after two or three weeks in a reservoir solution of 25% (w/v) PEG3350 and 0.1M HEPES (pH 7.5). After optimization, the best crystallization condition contained 0.08 μl protein (8–10 mg ml^−1^) in 20 mM Tris–HCl (pH 8.0), 50 mM NaCl with 0.32 μl reservoir solution of 22–28% (w/v) PEG 3350, 0.1M HEPES (pH 7.5) equilibrated at 293K. The crystals were transferred to the reservoir solution plus 20% (w/v) PEG400 as cryo-protectant before they were flash-cooled by plunging into liquid nitrogen. They were then stored in liquid nitrogen for later use in X-ray diffraction experiments.

### Data collection, structure determination and structure analysis

Diffraction data were collected at 100K at the Shanghai Synchrotron Research Facility beamline BL-17U or at Beijing Synchrotron Research Facility station 3W1A, China. Data were indexed and scaled with Xia2 implemented in the CCP4 suite (28,29). The data collection statistics are summarized in Table 1. The crystals of the DNA complex belong to space group P42_1_2, with unit-cell parameters of *a* = *b* = 117.4 Å, *c* = 65.8 Å. The structure was solved by molecular replacement using Phaser (30), with chain A of the MBP1 apo-structure (PDB entries 1MB1 and 1BM8, stripped of waters and ligands) used as the search model. The asymmetric unit contains two protein monomers and a DNA duplex with a solvent content of 32%. The model was subsequently improved by manual building in Coot (31) and further refined using PHENIX with TLS (Translation/Libration/Screw) restraints (32,33). Atomic coordinates and structure factors of PCG2–DBD were deposited in the PDB with the code 4UX5. Stereochemical validation of the model was performed with MolProbity (34). The model quality and refinement statistics are shown in Table 1.

**Table 1.** Data collection and refinement statistics

	PCG2–DBD
Wavelength (Å)	0.9792
Resolution range (Å)	51.6–2.44 (2.53–2.44)
Space group	P 4 21 2
Unit cell	117.4,117.4, 65.9, 90, 90,90
Total reflections	236114
Unique reflections	17560 (1712)
Multiplicity	13.4(14.0)
Completeness (%)	99.80 (100.00)
Mean I/sigma (I)	13.2(4.1)
Wilson B-factor	54.05
R-merge	0.145(0.942)
R-meas	0.151(0.978)
CC1/2	0.853(0.525)
CC*	0.96(0.83)
R-work	0.2219 (0.2723)
R-free	0.2858 (0.3712)
Number of atoms	2466
macromolecules	2385
ligands	
water	81
Protein residues	254
RMS (bonds)	0.007
RMS (angles)	1.31
Ramachandran favoured (%)	97
Ramachandran outliers (%)	0
Clashscore	6.31
Average B-factor	54.60
macromolecules	54.70
ligands	
solvent	51.70

Statistics for the highest-resolution shell are shown in parentheses.

Dimer interface analysis was performed with PISA (http://www.ebi.ac.uk/msd-srv/prot_int/pistart.html) (35). Sequence alignment was performed with Clustal (36) and the figure was prepared with Boxshade (http://www.ch.embnet.org/software/BOX_form.html). Figures containing structures were generated with PyMOL (PyMOL Molecular Graphics system, Version 1.3 Schrodinger, LLC).

## RESULTS

### Structure of the PCG2–DBD–DNA complex

The 2.44 Å structure of the PCG–DBD–DNA complex was solved by molecular replacement using a model of the MBP1 N-terminal domain apo-structure (PDB: 1MB1 or 1BM8). The data collection and refinement statistics are presented in Table 1. The asymmetric unit contains two protein molecules (monomer A and B) and one double-stranded (ds) DNA molecule (14bp, strands C and D). All nucleotides of the DNA and 229 amino acid residues (14–127 for monomer A and 14–128 for monomer B) were built in the model (Figure 1a, b, c; Supplementary Figure S1). Other residues, including the His tag, 1–13 at the N-terminus and 128–138 at the C-terminus, are not visible in the electron density map and are likely to be flexible or disordered.

Monomer A and B have a virtually identical conformation with an RMS derivation of only 0.7 Å for their main chains. Both of them comprise a discontinuous, anti-parallel β-barrel structure (β1–6) packed against a loosely associated bundle of four α helices (A–D) with the topology of secondary structures elements arranged in the order β1-β2-β3-β4-αA-αB-β5-β6-αC-αD (Figure 1a). The main difference between monomer A and B was in their C-termini. In monomer B, the C-terminus was turned towards the N-terminus and located close to the DNA binding site, whereas the residues from 121–128 in monomer A were located further from the DNA binding site (Figure 1d). This variation in conformation of the C-terminus is in agreement with the previous NMR model of MBP1 that suggested that the flexible C-terminal region could bind to DNA using different orientations (17).

Structural superposition of the MBP1–DBD and the PCG2–DBD–DNA complex revealed that the conformations of the secondary structures in the two DBDs are virtually identical and the two DBDs are highly similar (Figure 1d). The RMSD (Root Mean Square Deviation) between the main chains of the DNA-free structure of MBP1 and those of monomers A and B in the PCG2–DBD–DNA complex was 0.7 and 0.9 Å, respectively. However, locally the main chain of residues 23, 24, 59, 82 and 84 of monomer A or B in the complex undergo large shifts (>1.0 Å) compared with the MBP1 apo-structure. With the exception of residue 59, all of the other four residues are conserved between the members of the MBP1 protein family, including MBP1, Res1 and Res2 (Figure 1b), and are located in the DNA binding interface, Q82 and G84 in the 80 loop (the wing) and residues 23 and 24 in the 20-loop between β1 and β2 (Figure 1b).

In the complex, the MCB–DNA adopts a right-handed B-DNA. However, in the MCB–DNA as compared with the ideal B-DNA, major groove is narrower and the minor groove wider (Figure 1e). These differences likely result from the insertion of the 80-loop into the minor groove and further contacts by the 20-loop.

### Stoichiometry of the PCG2–MCB–DNA complex

**Figure 2.**
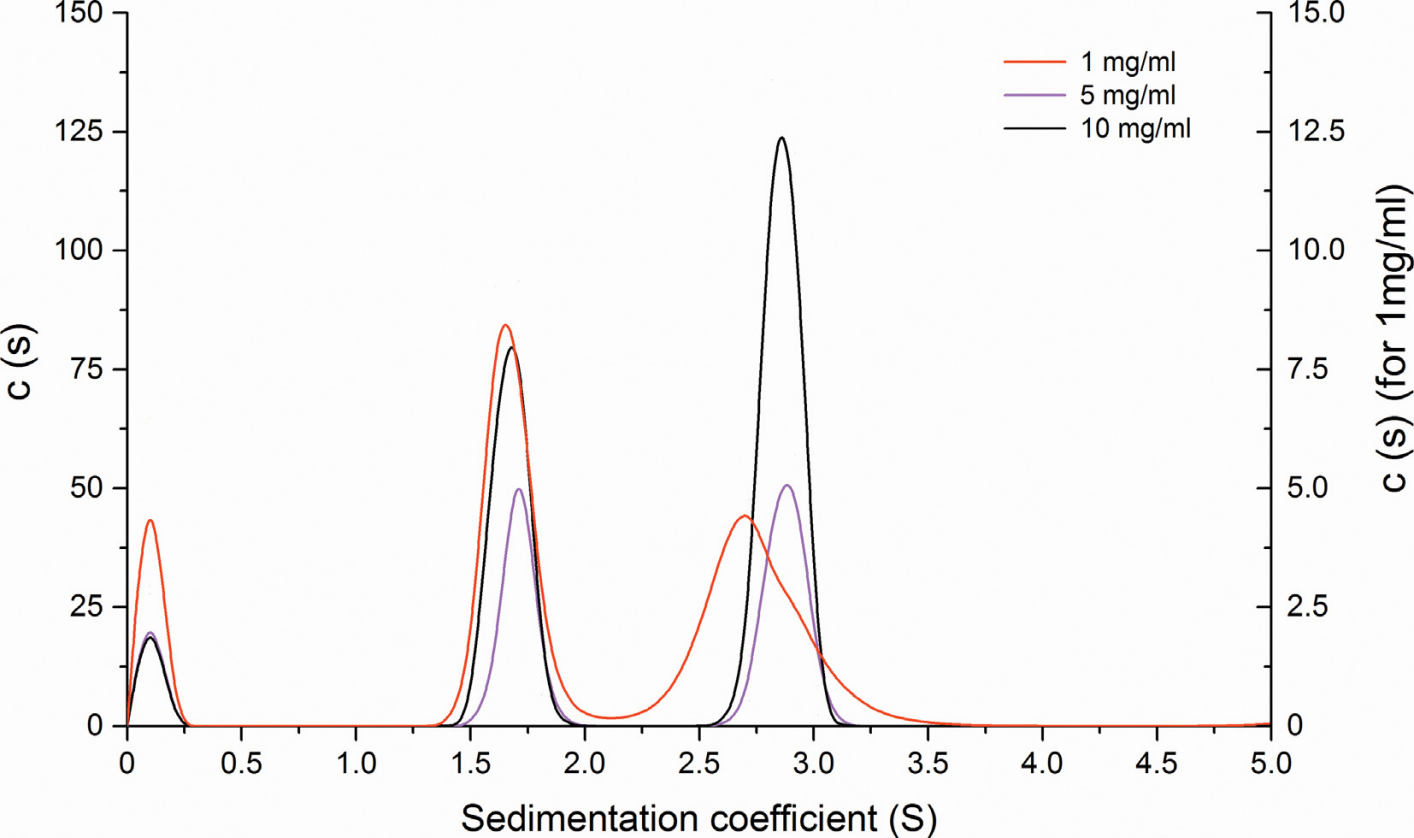
Characterization of the PCG2–DBD–DNA complex by AUC. Sedimentation velocity analysis. C(S) functions derived from sedimentation velocity data measured for PCG2–DBD–DNA complex samples at a fixed protein to DNA molar ratio od 2:1 and protein concentration of (1, 5, 10mg ml^−1^).

In the crystal structure of the protein–DNA complex, there are two PCG2–DBD molecules bound to one dsDNA suggesting an interaction stoichiometry of 2:1. This is different from the DNA and MBP1–DBD interaction, which was shown to be a 1:1 ratio in solution (19). Therefore, the stoichiometry of the DNA complex of PCG2–DBD was also analysed with SV-AUC using different protein concentrations ranging from 1 to 10 mg ml^−1^ whilst maintaining the molar ratio of protein to DNA ratio at 2:1. In the 1 mg ml^−1^ protein sample (∼60 μM), two major and one minor component with different sedimentation coefficients were apparent (Figure 2). The smallest component had a sedimentation coefficient of 1.7 S and molecular mass of 17 kDa, which is close to the expected molecular mass of one monomer of PGC2–DBD (16 kDa), is therefore likely the free PCG2–DBD. The second major component, which has a sedimentation coefficient of 2.7 S (molecular mass of ∼28 kDa), corresponds to a complex of one monomer of PCG2–DBD and DNA (expected molecular mass ∼25 kDa). In addition, at this low concentration there is also minor component (shoulder on the 2.7 S peak) with a sedimentation coefficient of 2.9 S. When the concentration of protein is increased to 5 or 10 mg ml^−1^, this larger 2.9 S corresponding to two monomers–DNA complex (molecular mass of ∼39 kDa) complex becomes predominant (Figure 2). These results demonstrate that PCG2–DBB can form different protein–DNA complexes in a concentration-dependent manner, and that at low concentration the 1:1 complex predominates but at higher concentration an additional protein can be accommodated onto the MCB–DNA.

### Protein–DNA interactions

**Figure 3.**
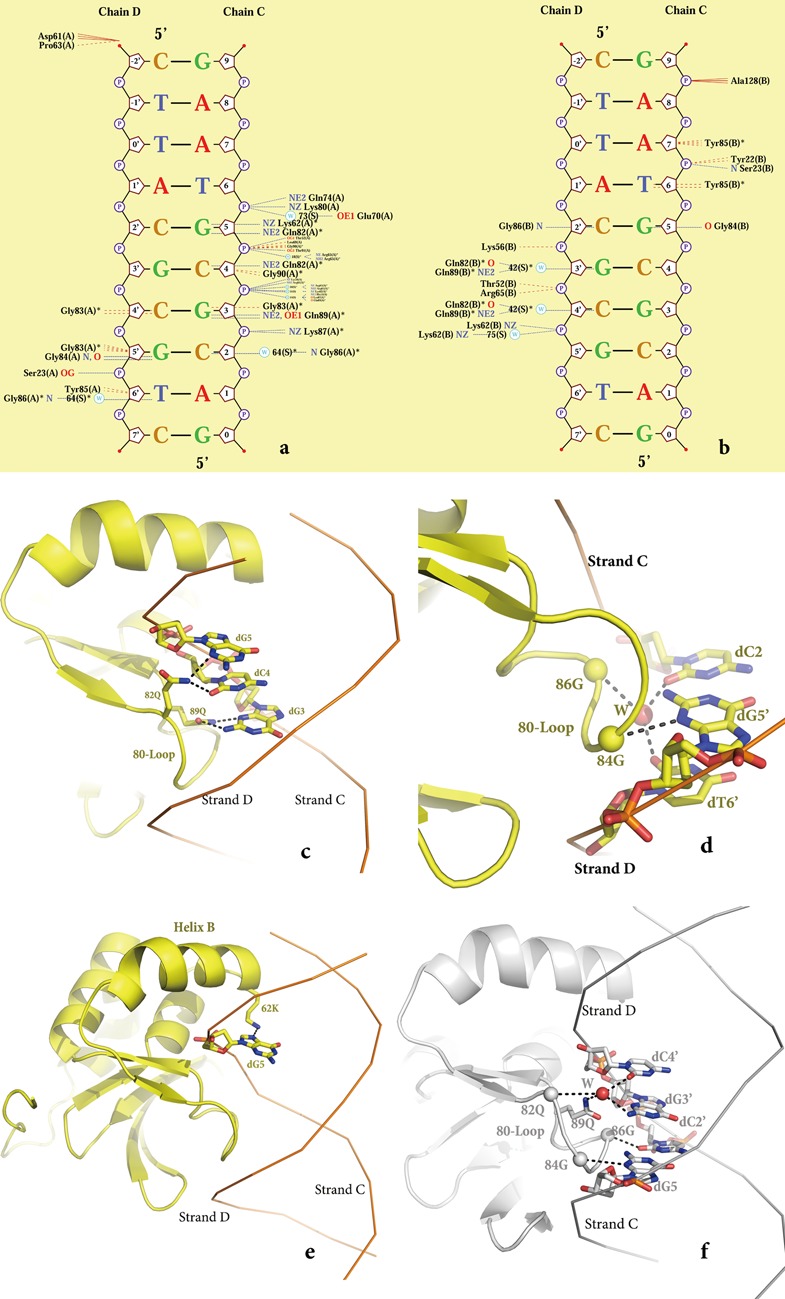
PCG2–DBD and MCB–DNA interactions. (**a**) and (**b**) Schematic illustrations (Ligplot) of interface 1 (a) and interface 2 (b) formed by monomers A and B, respectively. (**c**) Details of the contacts by Q82 and Q89 of monomer A that recognize the GCG of MCB element. (**d**) Interaction the central C of ‘ACGCGT’ with G86 of monomer A and recognition of G and T of the complementary strand ‘ACGCGT’ by G86 and G84 of monomer A. (**e**) Recognition of the G of ‘ACGCGT’ by K62 of monomer A. (**f**) Monomer B interface. Q82, Q89 and G86 make hydrogen-bonds with the central CGC of the MCB. G of the complementary strand ‘ACGCGT’ interacts with G84 of monomer B.

In the crystal structure of the PCG2–DBD–DNA complex, two protein molecules are bound to one dsDNA to form an ASU with two protein–DNA interfaces. The two interfaces are in close proximity covering the central CGCGT region of both strands of the MCD–DNA (Figure 3a, b; Supplementary Figure S2). Both interfaces incorporate extensive contacts between the protein monomers and the DNA. The total area of the interface between monomer A and the DNA (interface 1) was 1729.4 Å^2^, while the area between monomer B and the DNA (interface 2) was slightly smaller at 1330.0 Å^2^. However, the increased number of interactions, including hydrogen bonds and hydrophobic interactions, and larger DNA interface area formed in interface 1, indicate that the monomer-A conformation is likely the main conformation adopted by the PCG2–MCB protein–DNA interface.

For clarity and to precisely describe the protein–DNA interactions, the nucleobases of strand C (5′-CAATGACGCGTAAG-3′) are numbered from −4 to 9, making the core region ACGCGT 1–6, while the complementary strand D was numbered separately from −2′ to 11′ (5′-CTTACGCGTCATTG-3′) to make the core region ACGCGT numbered from 1′−6′ (Figure 1c). Inspection of protein–DNA interface 1 revealed that monomer A mainly forms hydrogen bonds with 2–6 (ACGCGT) of strand C and 5′-6′ (ACGCGT) of strand D in the MCB–DNA (Figure 3a). The residues that interact with DNA are located in the 20-loop, helices A and B, and the 80-loop (wing). In agreement with predicted and docking models of MBP1 (19,20), helix B is inserted into the major groove and makes numerous electrostatic contacts with the DNA and the wing (80-loop) makes contacts in the minor groove. By contrast, the 20-loop and helix A are novel elements in the winged-helix domains of PCG2 that non-specifically interact with DNA. These interactions include S23 in the 20-loop that directly interacts with the phosphate of dG5′ in strand D of the DNA and K56 and H53 in helix A that make salt bridges with the phosphate of dC4. Additionally, T51 and T91 interact with OP2 of dG5.

The conserved Glu-X-Gly-X-Gly-X-X-Glu (QXGXGXXQ) motif in the 80-loop is located in the minor groove of DNA and contains the residues that make the majority of protein-base interactions with the MCB–DNA. Three types of interactions are observed in this interface (Figure 3c and d). These include the side chain of Q89 that makes hydrogen bonds with the N2 and N3 of dG3 and the side chain of Q82 that is hydrogen bonded to the O2 of dC4 and the N3 of dG5. Notably, all three of these bases are found in the core MCB motif (GCG of ACGCGT) (Figure 3c). In addition, the main chain of G84 forms the second type of interaction with the dG5′ of strand D (Figure 3d) and in the third type of interaction, residue G86 interacts with the bases of dC2 and dT6′ through a water-mediated hydrogen bond (Figure 3d). In addition to these core interactions, K62 in helix B (Figure 3e) makes a hydrogen bond to the N7 of dG5 and along with R65 interacts with the phosphates of dC4 and dG5 through water-mediated binding (Figure 3a).

Interface 2 is formed between 5–7 (ACGCGTA) of strand C and 2′-5′ (ACGCGT) of strand D of the MCB–DNA with PCG2 monomer B (Figure 3b). The interacting residues are located in the same four secondary elements as those of monomer A. These include, two protein–phosphate interactions formed between the 20-loop and helix A with the DNA, S23 that interacts with the phosphate of dA7 of strand C and K62 that interacts with dG5′ of strand D both directly and indirectly through a water. Similarly to interface 1 all of the protein-base interactions are also formed between residues in the 80-loop and MCB–DNA in this interface (Figure 3f). These include the side chains of Q82 and Q89 that make hydrogen bonds with dG3′ and dC4′ of strand D through water, the main chain of G84 that interacts with dG5 and the complementary nucleotide dC2′ of strand D that interacts with the main chain of G86. As was observed in interface 1 all three of these bases are located in the core MCB motif (GCG of ACGCGT). However, hydrophobic interactions between G83 and G90 of monomer-A and the DNA in interface-1 (Figure 3a) are replaced by a hydrophobic interaction of Y85 of monomer-B with the DNA (Figure 3b).

### Structure-based functional analysis of the PCG2–DBD by SPR

To analyse the effect of mutations on the binding affinity of PCG2–DBD to MCB–DNA, mutant proteins with truncations (deletion of the N, C or both termini) or the single point mutations at key positions Q82 or Q89 were prepared. CD spectral analysis was employed to assess if the folding of the single point mutants was correct. The Q89L/N/Es or Q82L/N/Es mutant display a similar CD spectrum as the wild-type and were used for DNA binding assays (Supplementary Figure S3). Their DNA binding affinity was analysed by SPR (Biacore) analysis with MCB–DNA immobilized on the sensor chip. The results are presented in Table 2 and Supplemental Figure S4. The wild-type PCG2–DBD bound to MCB–DNA tightly, with an equilibrium dissociation constant (*K*_D_) of 0.81 μM. The N-terminal truncated mutant PCG2–DBD (12-138) bound to MCB–DNA with a *K*_D_ of 1.37 μM, which is similar to that of the wild-type, indicating that the N-terminal residues 1–11 have little or no effect on DNA binding. In contrast, deletion of the C-terminal region (residues 129-138) resulted in over a 30-fold decrease in the DNA binding affinity of the mutant with a *K*_D_ of 29.8 μM. When the Q82L or Q82N point mutations were introduced, the MCB binding affinity was also reduced at least 30-fold compared to that of the wild-type. When Q89L/N/E or Q82E substitutions were introduced, the binding activity was non-detectable. These data reveal that Q89, Q82 and residues 129–138, are key residues required for interaction of PCG2–DBD with DNA (Table 2; Supplementary Figure S4).

**Table 2.** Affinity constants for wild-type or mutant proteins binding to MCB–DNA

Protein	Binding affinity KD (μM)	Concentration range (μM)
WT	0.81 ± 0.03	0.125–8
Q82L	46 ± 4.87	1–128
Q82N	54 ± 2.7	1–128
12–138	1.37 ± 0.45	0.25–8
12–128	48.3 ± 5.2	2–128
1–128	29.8 ± 6.3	2–256

## DISCUSSION

### Key elements and residues of MBP1 homologous proteins that interact with DNA

In this study, we determined the crystal structure of PCG2–DBD–DNA complex. The structure revealed that the 80-loop (the wing) and helix B are key elements for DNA binding and interact with the minor and major grooves of the DNA at the protein–DNA interface. These data are consistent with previous predictions based on the apo-structure, NMR cross saturation experiments and mutational and docking analysis of structures of the yeast MBP1–DBD (12–15,17,19 and 20). Although the flexible C-terminus of PCG2–DBD could not be built in the model, the substantially weaker DNA binding of C-terminal deletion mutants in our binding assays demonstrated its importance in DNA binding (Supplementary Figure S4c). This C-terminal region is poorly conserved among MBP1 homologues (Figure 1b). However, it does contain several positively charged amino acids and in agreement with previous studies it is likely that although dynamically disordered this region contributes to the DNA–protein interface through non-specific electrostatic interactions (15). In contrast to the C terminus, deletion of the 11 N-terminal residues of PCG2, which are absent in the MBP1 of yeast, showed little effect on the DNA binding activity, suggesting that they were dispensable for DNA binding. Our data also demonstrate that two additional elements, the 20-loop and helix A, which were not predicted in the MBP1 apo-structure and MBP1–DNA interaction analysis (19,20), are also important for DNA binding. In addition, even the well-characterized DNA binding element, the 80-loop, has a distinct DNA binding property that had not been predicted in the previous reports (19,20).

Within the MCB-box recognized by MBP1 is the 2–5 segment (CGCG) core element in the centre of the consensus sequence 5′-ACGCGT-3′ and mutations in any of these core nucleotides severely diminish binding by MBP1(19,20). When single base pairs of the dsDNA–MCB1 have been systematically altered, changes in the core CGCG sequence show large difference in Gibbs energy of binding compared with the flanking region (19). In the structure, dC2 in the core element CGCG is recognized by the main chain of G86 through water and the complementary base dG5′ forms hydrogen bonds with the main chain of G84 (Figure 3a and e). Replacement of this CG2 pair with AT or TA would eliminate these hydrogen bonds and indeed abolishes the binding by MBP1 (19). A change of dG3 to dT will also affect the binding affinity because of the elimination of the hydrogen bond between dG3 and the side chains of Q89 (Figure 3a, c). A similar effect is observed when dG5 is substituted with dT, and the interactions between the base and the side chain of Q82 can no longer be formed. The importance of dC4 in the CGCG on recognition can be explained by the interaction between complementary base dG3′ and Q82 and Q89 from monomer B through water (Figure 3b and f). Our data now demonstrate that nucleotides within this CGCG element interact with residues in a conserved 82–89 (QXGXGXXQ) motif in the 80-loop through both side chain and main chain interactions. The two conserved glycines interact with DNA through non-specific main chain interactions while the two glutamines interact with DNA specifically through their side chains. Our structural and mutagenesis data reveal that among these conserved residues Q82 and Q89 are key for recognition of the central CGCG element and the equivalent Q67 in MBP1 (Q82 in PCG2) was also demonstrated to be an important residue for interacting with the minor groove of the target DNA (19). Notably, the N-terminal domain of Swi6 has a shorter 80-loop and also lacks the residues corresponding to the 20-loop (Supplementary Figure S5). These characteristics, together with the different electrostatic potential distribution of Swi6 (37), render the protein unable to bind to DNA.

In summary, two helices (A and B) and two loops (20 and 80 loops) of PCG2–DBD are major elements for DNA binding together with the flexible C-terminus and the conserved residues Q82 and Q89 in the 80-loop (wing) are key residues to recognize DNA.

### The wHTH of PCG2–DBD has a novel DNA binding mode

**Figure 4.**
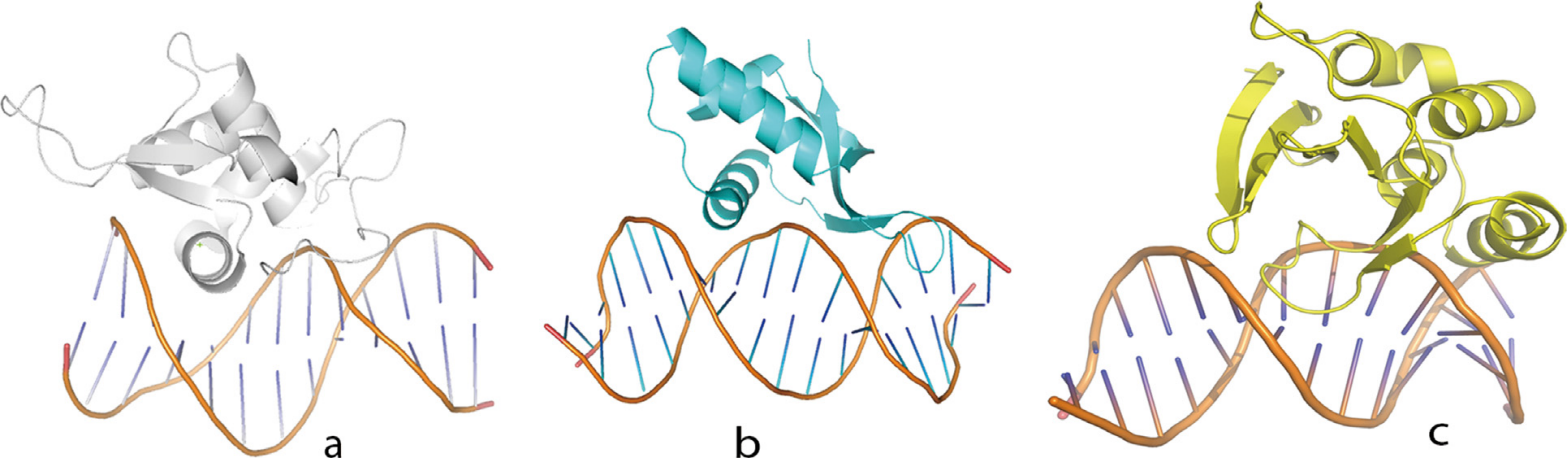
The DNA binding modes of wHTH domains. (**a**) HNF3γ has the canonical DNA binding mode where the recognition helix (helixB) interacts with the major groove of DNA by helix B. (**b**) RFX1 utilizes the second binding-mode where the wing binds in the major groove of dsDNA and helix B interacts with the the minor groove. (**c**) PCG2–DBD uses a new mode where the wing binds the minor groove of DNA and helix B interacts with the major groove of target DNA.

The wHTH is a well characterized nucleic acid binding domain that can interact with dsDNA, ssDNA or RNA in a variety of ways (38). To date, two different types of dsDNA binding modes have been described for wHTH domain (38,39; Figure 4a and b). One mode is the canonical (HNF3γ-like) DNA interaction profile, which is conserved in most wHTH-containing transcription factors. In this profile, transcription factor proteins interact with the major groove of DNA by employing helix B as the recognition helix (4,21–23 and 25; Figure 4a). In the other mode, utilized by the RFX1 transcription factor (24), it is the wing or hairpin that recognizes the major groove of dsDNA and helix B interacts with the minor groove (24, Figure 4b). In the PCG2 complex the wing of the wHTH domain binds to the minor groove, helix-B interacts with the major groove but with the wing making the majority of interactions at the centre of the protein–DNA interface (Figure 4c). Furthermore, it has four residues that form hydrogen bonds with DNA and three of the four also interact with the bases of two DNA strands at the core of the complex (Figure 3), indicating that the wing of PCG2–DBD is a key element in DNA recognition. In contrast with most wHTH structures, helix B is not the main mediator of DNA binding with only two residues (K62 and R65) making contacts with a base and a phosphate of the MCB–DNA.

Additionally, the two other conserved secondary structure elements (the 20-loop and helix A) are located on either sides of the wing and combine along with the C-terminal region of PCG2–DBD to increase its binding affinity. These data reveal how PCG2 uses the two glutamines Q82 and Q89 of the wing as the major recognition element to bind the core region CGCG of MCB–DNA. Moreover, the wing, 20 loop and Helix A are highly conserved in the MBP1 homologous proteins (Figure 1b), indicating that the DBD of all MBP1 homologues likely use this same third-mode mode of binding to recognize MCB elements.

### Different modes of PCG2–DNA complex formation

Most wHTH domains bind to their DNA targets as monomers. However, E2F/DP1 and RFX1 recognize DNA duplexes as dimers (4,24 and 39). Our AUC data supports the notion that one or two PCG2–DBDs can interact with a single MCB–DNA duplex (Figure 2), suggesting that 1:1 and 2:1 complexes can be formed in solution. In agreement with the AUC data, two monomers, A and B are bound to a single dsDNA to form a complex of two protein monomers and DNA in one asymmetric unit of crystal structure. A comparative analysis of the interactions of monomer A or B with dsDNA gives some clues to the nature of complex formation by the PCG2–DBD. Interface-1, formed in complex A, comprises a larger buried area than interface-2 in complex B (1729.4 Å^2^ versus 1330.0 Å^2^) and contains many more hydrogen bonds (26 versus 8) suggesting that there is a greater degree of specificity and affinity in the monomer-A interaction over the monomer-B interaction with DNA. When complex A and B was superposed with the core region of the MCB motif, helix B and half of the wing (80-loop) of monomer A align much closer to the DNA than their counterparts of monomer B while S23 of the 20-loop and G84, Y85 and G86 of the two monomers are in nearly the same positions to interact with DNA (Supplementary Figure S6). This movement of the 80-loop and helix-B, that decreases the interaction with DNA, may be caused by hindrance through lack of space for the 80-loop of monomer B to interact with DNA when the 80-loop of monomer-A is engaged with the DNA. One explanation for the presence of two modes of complex is that the complex A represents the specific PCG2–MCB interaction present in solution while monomer B represents the weaker complex, complex B that we observe only at high concentration. However, it remains unknown whether the 2:1 complex is present *in vivo* or if the weaker complex represents a more non-specific complex that might track the DNA until an MCB target sequence is encountered. Further biochemical and biophysical studies to investigate the interaction of MBP and PCG2 with MCB elements in a cellular context will be required to understand the *in vivo* prevalence and function of these specific and non-specific complexes.

## ACCESSION NUMBERS

PDB IDs: 4UX5, 1MB1 and 1BM8.

## SUPPLEMENTARY DATA

Supplementary Data are available at NAR Online.

SUPPLEMENTARY DATA
