# Supplementary material for: Structural basis of DNA recognition by PCG2 reveals a novel DNA binding mode for winged helix-turn-helix domains
Source: Nucleic Acids Res. 2014 Dec 29;43(2):1231–40. doi: 10.1093/nar/gku1351 (PMC4333399; doi:10.1093/nar/gku1351)
Supplement: SUPPLEMENTARY DATA [file supp_43_2_1231__index.html]

Structural basis of DNA recognition by PCG2 reveals a novel DNA binding mode for winged helix-turn-helix domains — SUPPLEMENTARY DATA 

# Structural basis of DNA recognition by PCG2 reveals a novel DNA binding mode for winged helix-turn-helix domains

## SUPPLEMENTARY DATA

**Files in this Data Supplement:**

- SUPPLEMENTARY DATA
- SUPPLEMENTARY DATA
- SUPPLEMENTARY DATA
- SUPPLEMENTARY DATA
