# Supplementary material for: Structural basis of DNA recognition by PCG2 reveals a novel DNA binding mode for winged helix-turn-helix domains
Source: Nucleic Acids Res. 2014 Dec 29;43(2):1231–40. doi: 10.1093/nar/gku1351 (PMC4333399; doi:10.1093/nar/gku1351)
Supplement: SUPPLEMENTARY DATA [file supp_gku1351_nar-02719-h-2014-File008.pdf]

Supplemental Figures

Figure S1. A 2Fo-Fc electron density map of the MCB DNA within the complex (blue mesh) contoured at 1.0σ.

Figure S2. The interfaces formed by the two monomers of PCG2-DBD with the MCB DNA strands C and D.

Figure S3. Far-UV circular dichroism analysis of wild-type and mutants of PCG2-DBD.

Figure S4. MCB DNA binding assays of the wild type(a) and mutants (b-g) of PCG2-DBD using Biacore T100: 1-128(b), 12-128 (c), 12-138 (d), Q82L (e) andQ82N (f). For mutants Q82E, Q89L, Q89N, and Q89E, due to the low affinity to DNA, the normalized response values of them and the wild type at 8 μM are shown in one panel (g).

Figure S5. Structure superposition of the PCG2-BDD DNA complex A (yellow) and the N-terminus of Swi6 from the budding yeast (blue).

Figure S6. Superposition of the structures of complexes A (yellow) and B (grey) on the core region of CGCG of "ACGCGT" to show the change in the 80-loop between monomer A and monomer B.

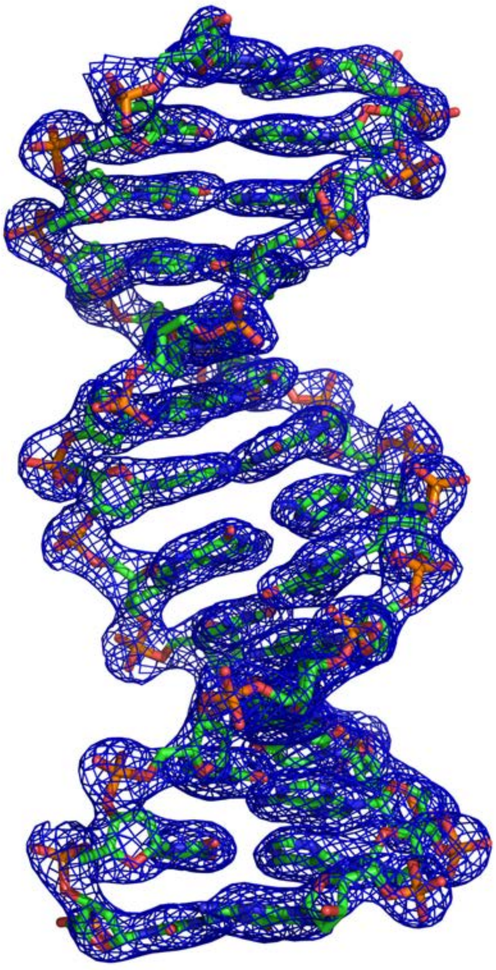

Figure S1 A 2Fo-Fc electron density map of the MCB DNA within the complex (blue mesh) contoured at 1.0σ

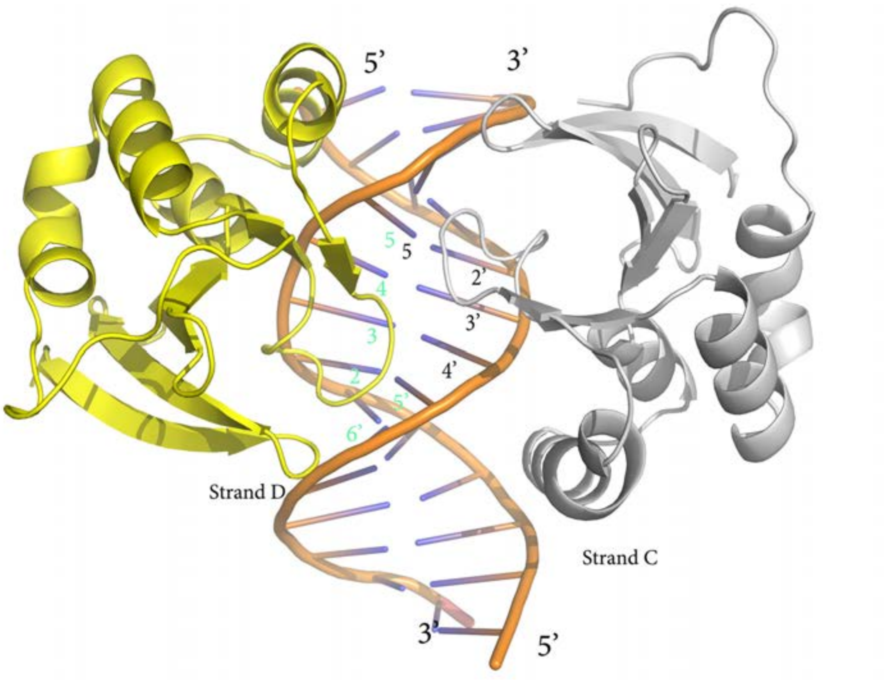

Figure S2 The interfaces formed by the two monomers of PCG2-DBD with the MCB DNA strands C and D.

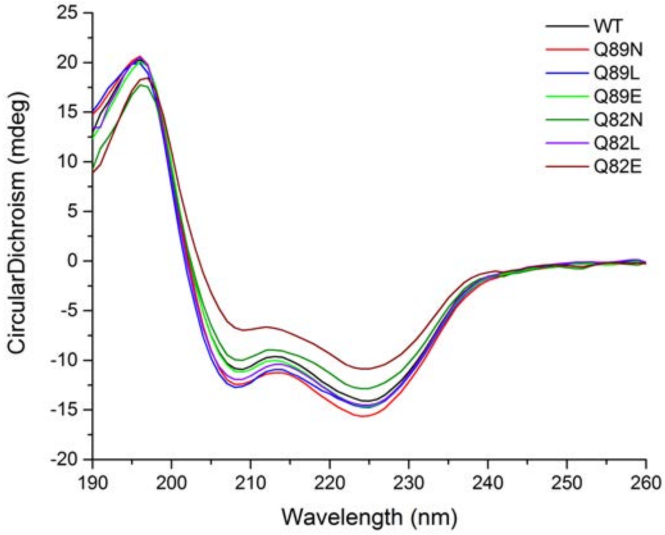

Figure S3 Far-UV circular dichroism analysis of wild-type and mutants of PCG2-DBD.

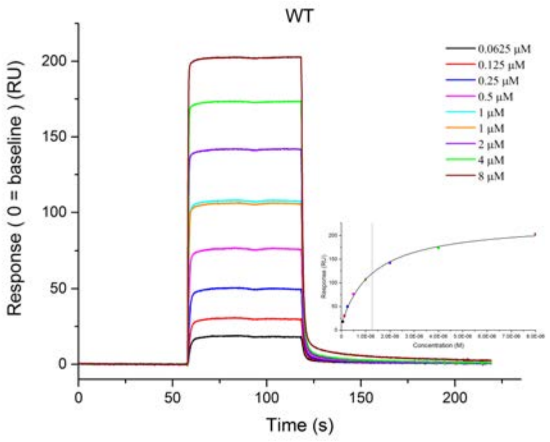

a

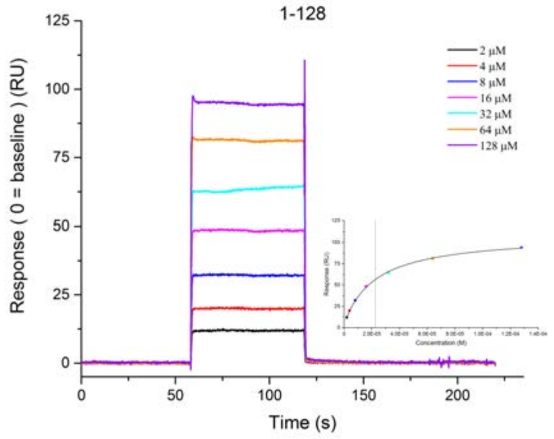

b

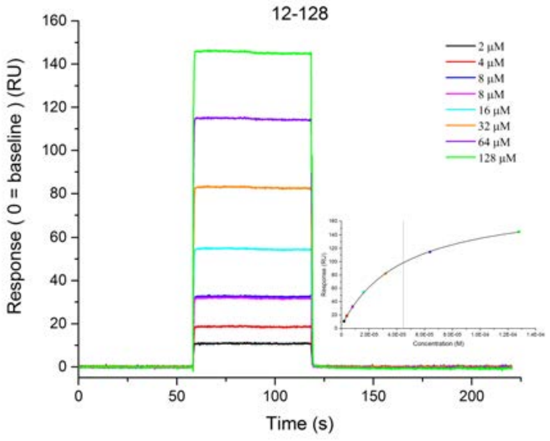

c

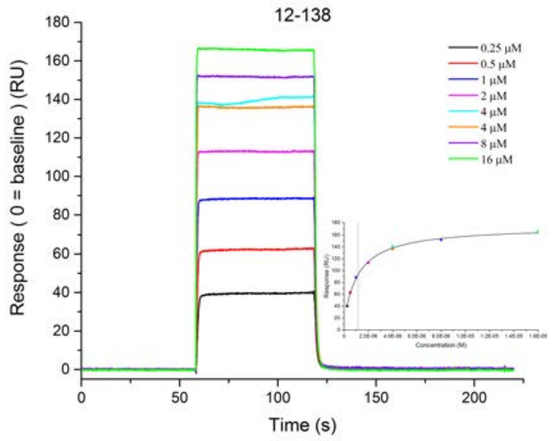

d

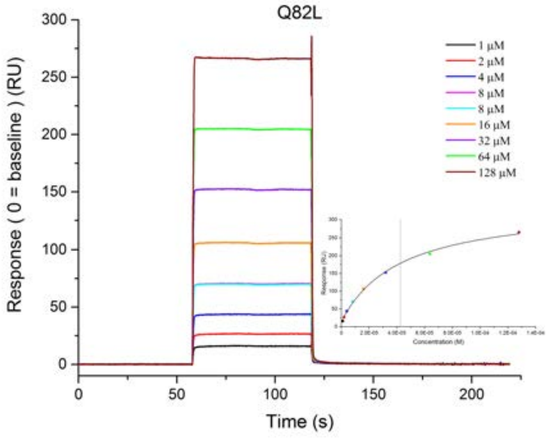

e

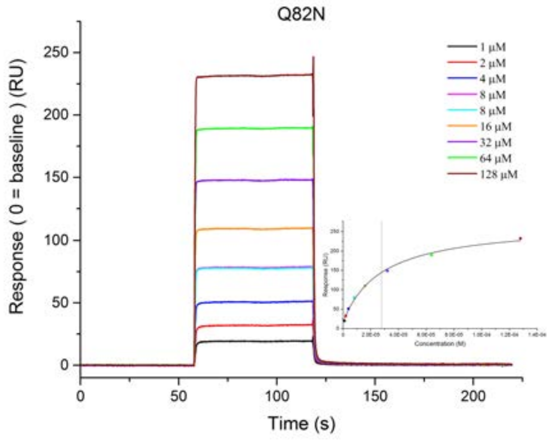

f

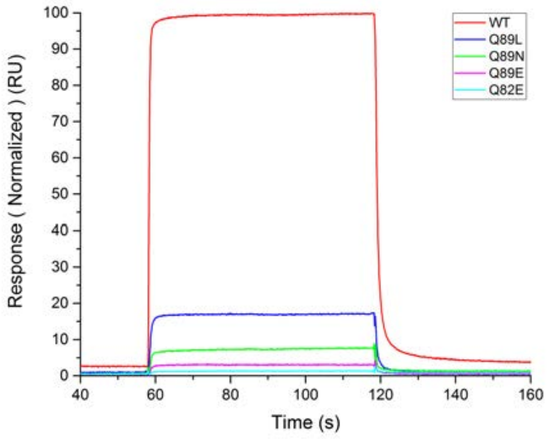

g

Figure S4 MCB DNA binding assays of the wild type(a) and mutants (b-g) of PCG2-DBD using Biacore T100: 1-128(b), 12-128 (c), 12-138 (d), Q82L (e) andQ82N (f). For mutants Q82E, Q89L, Q89N, and Q89E, due to the low affinity to DNA, the normalized response values of them and the wild type at 8 μM are shown in one panel (g).

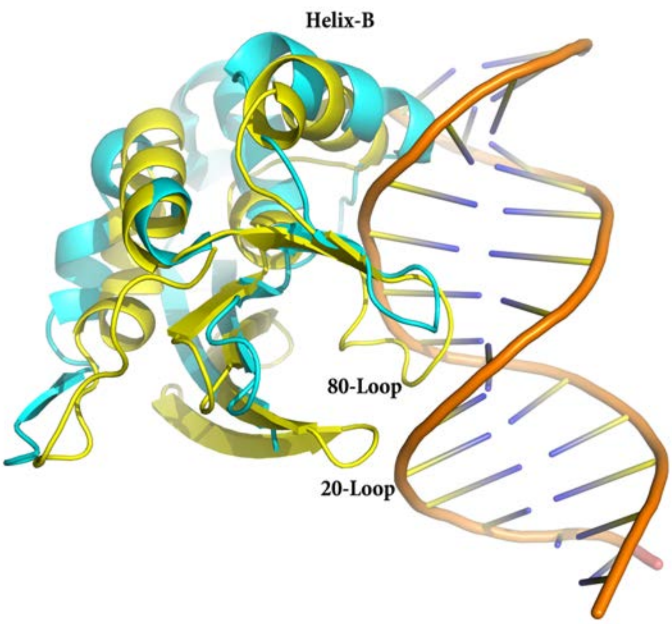

Figure S5 Structure superposition of the PCG2-BDD DNA complex A (yellow) and the N-terminus of Swi6 from the budding yeast (blue).

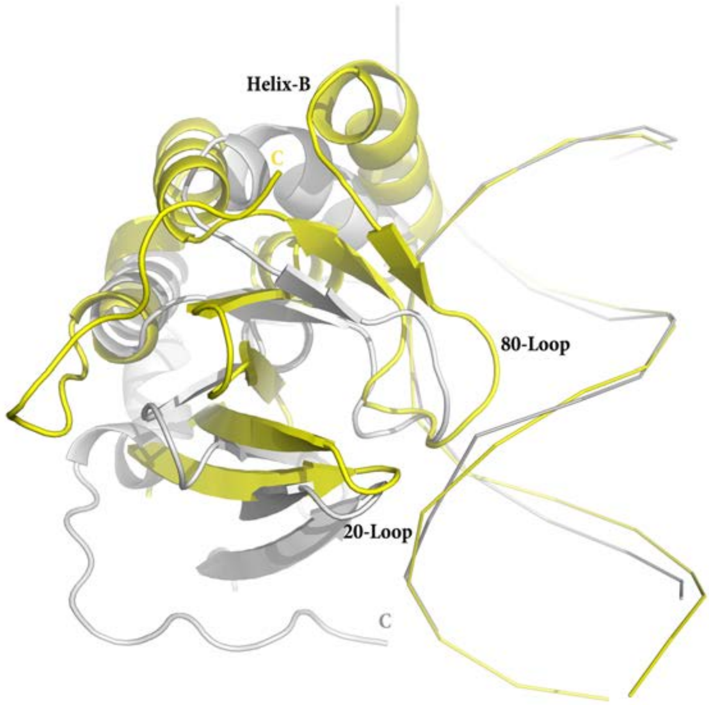

Figure S6 Superposition of the structures of complexes A (yellow) and B (grey) on the core region of CGCG of "A<u>CGCG</u>T" to show the change in the 80-loop between monomer A and monomer B.
