# Supplementary material for: Structural basis of DNA recognition by PCG2 reveals a novel DNA binding mode for winged helix-turn-helix domains
Source: Nucleic Acids Res. 2014 Dec 29;43(2):1231–40. doi: 10.1093/nar/gku1351 (PMC4333399; doi:10.1093/nar/gku1351)
Supplement: SUPPLEMENTARY DATA [file supp_gku1351_nar-02719-h-2014-File009.pdf]

EDS summary for PDB entry 4ux5.

The following statistics were obtained using the structure factors deposited for your PDB entry, using a customised version of the Uppsala Electron Density Server (EDS) running at PDBe. More information about EDS and the output presented here can be found on the original EDS site (http://eds.bmc.uu.se).

**Resolution**	
Resolution from map-calculations (low)	82.99Å
Resolution from map-calculations (high)	2.27Å
Resolution from PDB header	2.40Å
**R-factors**	
R-factor for map	0.279
R-factor from PDB header	0.231
Free R-factor from PDB header	0.258
**Structure quality**	
Average Real space R-factor (Deviation)	0.169 (0.045)
Average Real-space correlation coefficient (Deviation)	0.909 (0.082)
Average Occupancy-weighted avg temperature factor (Deviation)	52.4$Å^2$ (16.4$Å^2$)
**Wilson statistics**	
Wilson B-factor	49.5 $Å^2$
Wilson Scale	0.264
Wilson Omega	1.603
**Crystal data**	
Space group	P 4 21 2
Total no. of reflections	21702
Number of reflections used	21699
Completeness of data	99.8%

CONFIDENTIAL: VALIDATION REPORT

PQS summary for PDB entry 4ux5.

Assemblies are generated using the Protein Quaternary Structure server (PQS). PQS uses crystal symmetry matrices to generate symmetry-related copies of the chains in a PDB entry and, by considering the buried surface area between pairs of chains, determines the likelihood of that contact being of some biological significance. The algorithm is described in the PQS documentation on the PDBe website.

Biomolecule 1: Dimeric

Accessible surface areas	Isolated chain A (2 copies):	7905.0Å^2
	Total area of all isolated chains:	18903.2Å^2
	Total buried surface area of assembly:	-1546.6Å^2
Solvation free energies	Isolated chain A:	-100.7 kcal/mol
	Total energy for complete complex:	-200.1 kcal/mol
	Energy gain upon complex formation:	1.3 kcal/mol
Salt bridges	Number formed in assembly:	0

CONFIDENTIAL: VALIDATION REPORT

Sequence-matching information for PDB entry 4ux5.

We have matched your sequence for chain/s: A B C D ... to the following UniProt Reference and alignment. Please comment if this is correct. UniProt represents the combined TREMBL,SWISSPROT and PIR sequence databases. More information about UniProt may be found at the Uniprot website (http://www.uniprot.org).

INFORMATION FOR CHAIN A

UNP ACCESSION NUMBER: G4NA99
UNP ORGANISM SCIENTIFIC: MAGNAPORTHE ORYZAE
TAX ID: 318829
PFAM ACCESSION NUMBER(S): PF13637, PF00023, PF04383
UNP SEQUENCE START-END POSITION(S): 1 - 138
UNP-PDB ATOM RECORDS ALIGNMENT:

```
UNP              1 MVKAAAAAASAPTGPGIYSATYSGIPVYEYQFGVDLKEHVMRRRVDDWIN    50
                   |||||||||||||||||||||||||||||||||  |||||||||||||||
PDB              1 MVKAAAAAASAPTGPGIYSATYSGIPVYEYQFG--LKEHVMRRRVDDWIN    48

UNP             51 ATHILKAAGFDKPARTRILEREVQKDQHEKVQGGYGKYQGTWIPLEAGEA   100
                   ||||||||||||||||||||||||||||||||||||||||||||||||||
PDB             49 ATHILKAAGFDKPARTRILEREVQKDQHEKVQGGYGKYQGTWIPLEAGEA    98

UNP            101 LAHRNNIFDRLRPIFEFSPGPDSPPPAPRHTSKPKQPKKPAVPRFNNKAR   150
                   ||||||||||||||||||||||||||||||||||||||
PDB             99 LAHRNNIFDRLRPIFEFSPGPDSPPPAPRHTSKPKQPK------------   136

UNP            151 AVAKQAPPPPLHYQPSLHPQDAYENGEMLVDEDDTPDNLTVASASYMAED   200
PDB            137 --------------------------------------------------   136

UNP            201 DRPDLSHFSTGHRKRKREEPTESMIEQQHRIYGDELLDYFLLSRNQPTPA   250
PDB            137 --------------------------------------------------   136

UNP            251 MRPEPPVNFRPNFPIDADQHTALHWAASMGDVDIIKQLFQFNAQPDSRNV   300
PDB            137 --------------------------------------------------   136

UNP            301 RGETPLMRAVTFTNCYDKQTFPVVLKELFHTINIRDLSGCTAIHHAAILK   350
PDB            137 --------------------------------------------------   136

UNP            351 GGRVHSPTCSRYYLDNILNRLQETQHDPNFVQQLLDAQDNDGNTAVHLAA   400
PDB            137 --------------------------------------------------   136

UNP            401 QRGSSKCIRALLGRGASTDITNNEGMIAADLIKELNASKKLRSVPQRSSS   450
PDB            137 --------------------------------------------------   136

UNP            451 PFAPESARRQVSFRDALAGDVGAMTIGAANSHSNKMTASLKSEAALTVQN   500
PDB            137 --------------------------------------------------   136

UNP            501 RITPLVFEKFHDLARSYEDEFTIKDEAEREATRILSNAQAEHTSLTNKLA   550
PDB            137 --------------------------------------------------   136

UNP            551 ELGSQLLPPEQAFGIDGELETATGKVKAVVCTLNRLHVEGLVERELDASM   600
PDB            137 --------------------------------------------------   136

UNP            601 NGDSNGNTNSIEERLALANELRYLLGEQQMAETEYIEALSMVGTGEKIDQ   650
PDB            137 --------------------------------------------------   136

UNP            651 YRRLLRNCLGPLAENLDDNLEELVAIMEEEVSDGMVNSTADGPSSVTNGL   700
PDB            137 --------------------------------------------------   136

UNP            701 VGGETMDLAPPTSAL    715
PDB            137 ---------------    136
```

INFORMATION FOR CHAIN B

EMBL-EBI PDBe PROTEIN DATA BANK EUROPE
CONFIDENTIAL. VALIDATION REPORT

UNP ACCESSION NUMBER: G4NA99
UNP ORGANISM SCIENTIFIC: MAGNAPORTHE ORYZAE
TAX ID: 318829
PFAM ACCESSION NUMBER(S): PF13637, PF00023, PF04383
UNP SEQUENCE START-END POSITION(S): 1 - 138
UNP-PDB ATOM RECORDS ALIGNMENT:

```
UNP                   1 MVKAAAAAASAPTGPGIYSATYSGIPVYEYQFGVDLKEHVMRRRVDDWIN     50
                        |||||||||||||||||||||||||||||||||  |||||||||||||||
PDB                   1 MVKAAAAAASAPTGPGIYSATYSGIPVYEYQFG--LKEHVMRRRVDDWIN     48

UNP                  51 ATHILKAAGFDKPARTRILEREVQKDQHEKVQGGYGKYQGTWIPLEAGEA    100
                        ||||||||||||||||||||||||||||||||||||||||||||||||||
PDB                  49 ATHILKAAGFDKPARTRILEREVQKDQHEKVQGGYGKYQGTWIPLEAGEA     98

UNP                 101 LAHRNNIFDRLRPIFEFSPGPDSPPPAPRHTSKPKQPKKPAVPRFNNKAR    150
                        ||||||||||||||||||||||||||||||||||||||
PDB                  99 LAHRNNIFDRLRPIFEFSPGPDSPPPAPRHTSKPKQPK------------    136

UNP                 151 AVAKQAPPPPLHYQPSLHPQDAYENGEMLVDEDDTPDNLTVASASYMAED    200
PDB                 137 --------------------------------------------------    136

UNP                 201 DRPDLSHFSTGHRKRKREEPTESMIEQQHRIYGDELLDYFLLSRNQPTPA    250
PDB                 137 --------------------------------------------------    136

UNP                 251 MRPEPPVNFRPNFPIDADQHTALHWAASMGDVDIIKQLFQFNAQPDSRNV    300
PDB                 137 --------------------------------------------------    136

UNP                 301 RGETPLMRAVTFTNCYDKQTFPVVLKELFHTINIRDLSGCTAIHHAAILK    350
PDB                 137 --------------------------------------------------    136

UNP                 351 GGRVHSPTCSRYYLDNILNRLQETQHDPNFVQQLLDAQDNDGNTAVHLAA    400
PDB                 137 --------------------------------------------------    136

UNP                 401 QRGSSKCIRALLGRGASTDITNNEGMIAADLIKELNASKKLRSVPQRSSS    450
PDB                 137 --------------------------------------------------    136

UNP                 451 PFAPESARRQVSFRDALAGDVGAMTIGAANSHSNKMTASLKSEAALTVQN    500
PDB                 137 --------------------------------------------------    136

UNP                 501 RITPLVFEKFHDLARSYEDEFTIKDEAEREATRILSNAQAEHTSLTNKLA    550
PDB                 137 --------------------------------------------------    136

UNP                 551 ELGSQLLPPEQAFGIDGELETATGKVKAVVCTLNRLHVEGLVERELDASM    600
PDB                 137 --------------------------------------------------    136

UNP                 601 NGDSNGNTNSIEERLALANELRYLLGEQQMAETEYIEALSMVGTGEKIDQ    650
PDB                 137 --------------------------------------------------    136

UNP                 651 YRRLLRNCLGPLAENLDDNLEELVAIMEEEVSDGMVNSTADGPSSVTNGL    700
PDB                 137 --------------------------------------------------    136

UNP                 701 VGGETMDLAPPTSAL    715
PDB                 137 ---------------    136
```

CONFIDENTIAL: VALIDATION REPORT

Deviations from expected geometric properties (REMARK 500) for PDB entry 4ux5.

```
REMARK 500
REMARK 500 GEOMETRY AND STEREOCHEMISTRY
REMARK 500 SUBTOPIC: TORSION ANGLES
REMARK 500
REMARK 500 TORSION ANGLES OUTSIDE THE EXPECTED RAMACHANDRAN REGIONS:
REMARK 500 (M=MODEL NUMBER; RES=RESIDUE NAME; C=CHAIN IDENTIFIER;
REMARK 500 SSEQ=SEQUENCE NUMBER; I=INSERTION CODE).
REMARK 500
REMARK 500 STANDARD TABLE:
REMARK 500 FORMAT:(10X,I3,1X,A3,1X,A1,I4,A1,4X,F7.2,3X,F7.2)
REMARK 500
REMARK 500 EXPECTED VALUES: GJ KLEYWEGT AND TA JONES (1996). PHI/PSI-
REMARK 500 CHOLOGY: RAMACHANDRAN REVISITED. STRUCTURE 4, 1395 - 1400
REMARK 500
REMARK 500  M RES CSSEQI        PSI       PHI
REMARK 500    ASP A  47       19.30     59.00
REMARK 500    GLU A  72      -60.87   -131.00
REMARK 500    GLU B  72      -60.74   -124.91
REMARK 500
REMARK 500 REMARK: NULL
```

CONFIDENTIAL: VALIDATION REPORT

EMBL-EBI PDBe PROTEIN DATA BANK EUROPE
